# Applications of Protein Microarrays in Biomarker Discovery for Autoimmune Diseases

**DOI:** 10.3389/fimmu.2021.645632

**Published:** 2021-05-03

**Authors:** Siting Li, Guang Song, Yina Bai, Ning Song, Jiuliang Zhao, Jian Liu, Chaojun Hu

**Affiliations:** ^1^ Department of Rheumatology, Peking Union Medical College Hospital, Peking Union Medical College & Chinese Academy of Medical Sciences, Key Laboratory of Rheumatology & Clinical Immunology, Ministry of Education, Beijing, China; ^2^ Department of Rheumatology, National Clinical Research Center for Dermatologic and Immunologic Diseases (NCRC-DID), Beijing, China; ^3^ Department of Pharmacology and Molecular Sciences, Johns Hopkins University School of Medicine, Baltimore, MD, United States; ^4^ Department of Rheumatology, Aerospace Center Hospital, Aerospace, Clinical Medical College, Peking University, Beijing, China

**Keywords:** protein microarray, proteome, biomarker discovery, autoimmune disease, experimental design

## Abstract

Dysregulated autoantibodies and cytokines were deemed to provide important cues for potential illnesses, such as various carcinomas and autoimmune diseases. Increasing biotechnological approaches have been applied to screen and identify the specific alterations of these biomolecules as distinctive biomarkers in diseases, especially autoimmune diseases. As a versatile and robust platform, protein microarray technology allows researchers to easily profile dysregulated autoantibodies and cytokines associated with autoimmune diseases using various biological specimens, mainly serum samples. Here, we summarize the applications of protein microarrays in biomarker discovery for autoimmune diseases. In addition, the key issues in the process of using this approach are presented for improving future studies.

## Introduction

Autoimmune diseases (AIDs) are a group of diseases characterized by disordered stimulation of self-reactive immune response attacking one's own organ or tissues. Autoantibodies play a vital role in the pathogenesis of AIDs and have been tested in clinic as biomarkers for diagnosis, prognosis, as well as patient stratification (1). In addition, alteration of cytokines, chemokines, protein glycosylation, and other molecules also provides bundles of valuable information to evaluate the progress of AIDs. However, the sensitivity and specificity of some routine tests (e.g., Antinuclear antibodies) cannot satisfy the diagnosis requirement for some common AIDs such as systemic lupus erythematosus (SLE), systemic sclerosis (SS), and rheumatoid arthritis (RA). Furthermore, there are no specific biomarkers for some AIDs, especially the ones with low incidence. More recently, protein microarray technology has attracted increasing attention from scientists working to discover novel biomarkers in various diseases. Compared to traditional techniques such as mass spectrometry, protein microarray technology is a robust and versatile platform that has the benefit of requiring only small quantities of protein to simultaneously survey a large variety of analytes in crude samples (2). For this reason, protein microarrays are a highly useful tool in biomarker discovery.

Protein microarray technology was inspired by the previously developed DNA microarray technology, which immobilizes hundreds of thousands of oligonucleosides onto a small glass slide and provides a high-throughput method to study the alteration of gene expression in cells or tissues. Similarly, protein microarrays are constructed by immobilizing large numbers of various proteins, peptides, antibodies, lectins, cell or tissue lysates. Based on the application, protein microarrays can be categorized as either an analytical array or a functional array (3). Usually, an analytical array is constructed with well-characterized affinity reagents such as antibodies to detect and quantify specific proteins (4). Functional arrays are assembled with purified recombinant proteins or peptide fragments and can be used in discovery-based studies, including studying protein-protein, protein-lipid, protein-DNA, protein-drug, and protein-peptide interactions (5). Alternatively, protein probes could also be printed onto a planar or bead-based platform (6). Based on the probes utilized on the array, protein microarrays can be categorized into antigen arrays, antibody arrays, peptide arrays, lectin array, etc. In addition to the precast recombinant proteins or cell lysates, protein probes could be synthesized *in situ* using cell-free expression systems based on printed plasmid DNA. Protein arrays assembled by this strategy are called Nucleic Acid-Programmable Protein Arrays (NAPPAs) (7). To date, a variety of both lab-made and commercial microarrays have been widely used to identify potential biomarkers for various diseases. In this review, we will focus on the application of protein microarrays in AID biomarker discovery, as well as the key issues that are often encountered in the process of using protein microarrays. An overview of the various protein microarrays that are commonly utilized is listed in **Table 1** and their applications with various AIDs are summarized in **Table 2**.

**Table 1:** Major protein microarray platforms for biomarker discovery in autoimmune diseases.

Type	Product name	Protein content	Company/Laboratory	Major applications in AID biomarker discovery	References
Antigen array	ProtoArray Human Protein Microarray	9483 unique full-length human proteins	Invitrogen	RA, IBD, type-1 diabetes, ankylosing spondylitis, primary Sjögren’s syndrome, ADEM, Meniere’s disease, neuromyelitis optica, myasthenia gravis, chronic renal disease, APS1, SLE, ulcerative colitis	(8, 9)
	HuProt Human Proteome Microarray	19,394 full-length human proteins	CDI Laboratories	SLE, MS, type-1 diabetes, PBC, dermatomyositis, Behcet disease, autoimmune hepatitis, neuropsychiatric lupus	(10, 11)
	Immunome Protein Array	1,636 full-length human proteins involved in the immune response	Sengenics	SLE, RA	(12, 13)
	Recombinant human protein microarray	E. coli expressed proteins from 37,200 human fetal brain cDNA	Horn laboratory	alopecia areata, dilated cardiomyopathy	(14, 15)
	UNIarray	3101 proteins or protein fragments from fetal brain cDNA	Protagen Diagnostics	MS	(16)
	Recombinant human protein microarray	1626 transmembrane and secreted proteins through *in silico* selection	Grifantini laboratory	autoimmune hepatitis. PBC	(17, 18)
	Nucleic Acid Programmable Protein Arrays (NAPPA)	~2500 in situ expressed protein	BioDesign	osteoarthritis, type-1 diabetes, ankylosing spondylitis, juvenile arthritis	(19, 20)
	Phage arrays	T7 phage display cDNA array biopanned by patient sera	Lin laboratory, D’Angelo laboratory	celiac disease, sarcoidosis	(21, 22)
	HPA arrays	Various number of protein fragments from Human Protein Atlas	Nilsson laboratory, Uhlen laboratory, etc.	MS, osteoarthritis, sarcoidosis, SLE	(23–25)
	RepliTope^™^ peptide microarray	10,000 random-sequence 20-mer peptides	JPT Peptide Technologies	SLE	(26)
Antibody array	Raybiotech cytokine antibody arrays	Antibodies against various human cytokines	RayBiotech	SLE, pre-eclampsia, Crohn’s disease	(27, 28)
	Bead-based LUNARIS™ BioChip	Antibodies against several key inflammatory biomarkers	AYOXXA	Sjogren’s Syndrome	(29)
	DotScan™ antibody microarray	82 mouse monoclonal antibodies against human CD antigens	Medsaic	SLE	(30)
Lectin array	Various lectin arrays	Lectins that binds glycol-biomarkers	Dang laboratory, Takeshita laboratory	IBD, RA	(31, 32)

**Table 2:** Selective application of protein microarrays in biomarker discovery for autoimmune diseases.

Diseases	Array type	Array platform	Sample type	Key findings	References
**Systemic lupus erythematosus**	Antigen array	ProtoArray	Serum	Over 300 novel autoantibodies were observed, a group of which concerning apoptosis was validated by ELISA and western blot, and positive correlation with anti-dsDNA was also found in SLE patients.	(33)
	Antigen array	HuProt Array	Serum	Four candidates were observed from the array, of which anti-CLIC2 was verified by ELISA and show significant result.	(34)
	Antigen array	HuProt Array	Cerebrospinal fluid	Microarray elicited 137 autoantigens correlating with neuropsychiatric systemic SLE, pathway and other analysis revealed association of several candidates with clinical manifestations.	(35)
	Antigen array	Immunome Protein Array	Serum	Totally 79 novel and previously-reported autoantigens were found, and analysis revealed 4 subgroup clusters related to 4 subgroups of SLE patients. A panel of 26 autoantigens show increased diagnostic accuracy.	(36)
	Antigen array	Lab-made array containing 140 recombinant or purified antigens	Serum	Fifty autoantibodies were significantly higher in sera of pediatric SLE patients compared to healthy controls, including anti-B cell-activating factor (BAFF) which was associated with active disease.	(37)
	Antigen array	silicon-based peptide microarrays	Serum	Microarrays with >5700 features corresponding to 843 unique peptides derived from the U1-70K protein identified multiple reactive epitopes, which was examined by indirect and competitive ELISA.	(38)
	Antigen array	Antigenic epitopes peptide array	Serum	Epitopes prediction by DNA star software were constructed into peptide array, and 14 epitopes with potential diagnostic values were screened out with high sensitivity and specificity for SLE.	(39)
	Antibody array	DotScan™ antibody microarray	Serum leukocytes	Microarrays containing mouse monoclonal antibodies against human CD antigens was used to profile SLE patients and a computational algorithm analysis assisted in distinguishing active SLE patients.	(30)
**Multiple sclerosis**	Antigen array	HuProt Array and PrEST array	Serum	PrEST array was fabricated, analyzed by antibody off-target interactions, and used for antibody profiling in secondary progressive MS patients, which was also conducted using HuProt Array.	(23)
	Antigen array	UNIarray	Cerebrospinal fluid	Ten novel antoantigens were identified to be specifically related to MS patients.	(16)
	Antigen array	PrEST array	Plasma	Prominently increased autoantibody reactivity against the chloride channel protein anoctamin 2 (ANO2) was observed in MS cases compared with controls.	(40)
	Antigen array	Peptide array	Serum and cerebrospinal fluid	Peptides deduced from 45 candidate proteins were concentrated into peptide microarray, of which 54 were associated with MS, and EV-virus related	(41)
**Rheumatoid arthritis**	Antigen array	ProtoArray	Serum	Four antigens were recognized almost uniquely by sera from patients with RA on protein arrays.	(42)
	Antigen array	Immunome Protein Array	Serum	A total of 102 proteins recognized by IgG autoantibodies were identified, of which 86 by antibodies were from CCP-positive RA patients and 76 were from anti-CCP-negative RA patients.	(13)
	Antigen array	Lab-made cell-free expression array	Serum and synovial tissue extract	Array antigens were screened with the AlphaScreen method, and antibodies against two proteins were visualized in the cytoplasm of plasmacytes in two RA synovitis lesions.	(43)
	Antibody array	Lab-made bead-based cytokine array	Serum	Multiplex measurement of 3 differentiating biomarkers provided high sensitivity and specificity in the diagnostic discrimination of RA.	(44)
	Lectin array	Lab-made lectin array	Serum and synovial fluid	Lectin array was utilized for glycosylation profiling of MMP-3, and 3 lectins were found correlated with RA.	(32)
**Systemic sclerosis**	Antigen array	SeroTag bead-based array	Serum	A set of 100–150 autoantigens, half of them well established, the other half novel, succeed in differential diagnosis of AID including SLE, SSc, RA, etc.	(45)
	Antigen array	centromere protein array	Serum	Statistical analysis revealed 11 CENP are potential target antigens of ACA in patients with SSc, among which CENP-P and CENP-Q showed high sensitivities.	(46)
	Antibody array	Recombinant ScFv array	Serum	Biomarker signatures differentiating SLE versus SSc were demonstrated and differences increased with severity of SLE, while serum profiles of SSc versus healthy controls were more similar.	(47)
**Juvenile arthritis**	Antigen array	NAPPA array	Plasma and synovial fluid	A strong correlation was observed for the levels of antibodies between plasma and synovial fluid in JIA patients, and 18 antigens were identified.	(48)
**Osteoarthritis**	Antigen array	PrEST array and NAPPA array	Serum	PrEST array revealed 373 antigens which were selected for validation on bead-based arrays, 80 of which were constructed into NAPPA array and validated by ELISA, and 9 and 7 osteoarthritis-related autoantibodies were confirmed respectively.	(49)
**Pre-eclampsia**	Antibody array	Raybio membrane-based cytokine array	Serum	sTNF-R1, Axl, and TIMP-2 were found elevated in patients with preeclampsia compared to gestational hypertensive patients and health pregnant controls.	(50)
**Chronic fatigue syndrome**	Antigen array	EBV-derived peptide microarray	Serum	Significantly enhanced IgG responses to several EBNA-6 peptides containing a repeat sequence homology to various human proteins were found in CFS patients compared to controls.	(51)
**Inflammatory bowel disease**	Antigen array	ProtoArray	Serum	Array analysis revealed 66 candidate antigens in patients with UC, of which 6 were validated using AlphaLISA technique.	(52)
	Antigen array	E. coli proteome array	Serum	Protein array containing 4,256 E. coli K12 proteins identified two sets of serum antibodies that were novel biomarkers for specifically distinguishing CD from healthy controls and UC.	(53)
	Lectin array	Lab-made lectin array	Serum	Two lectins had higher affinity for serum agalactosyl IgG from IBD patients, especially those with CD, compared to health controls.	(54)
	Antibody array	Raybio Cytokine array	Rectum and anus biopsy sample supernatant	Post-trauma cytokine profile was examined in CD patients.	(27)
**Type-1 diabetes**	Antigen array	ProtoArray	Serum	Anti-EEF1A1 and anti-UBE2L3 were selected from microarrays and validated by immunofluorescence staining of pancreas and ELISA.	(55)
	Antigen array	Viral antigen array	Serum	Antibody profiling of T1D patients on viral protein array comprising the complete proteomes of seven viruses indicated significant association between T1D and EBV virus.	(56)
	Antigen array	NAPPA array	Serum	NAPPA array containing ~1000 proteins identified six specific novel T1D-associated autoantibodies valeted by immunohistochemistry.	(57)
**Sarcoidosis**	Antigen array	Phage display array	Serum	Microarray constructed from T7 phage display cDNA library biopanned by sarcoidosis patients’ sera identified 50 clones that distinguished between TB, sarcoidosis, and healthy controls.	(21)
	Antigen array	PrEST array	Bronchoalveolar lavage and serum	PrEST array containing 3072 protein fragments identified a set of 131 targets that was subsequently verified on suspension bead arrays to elicit 4 sarcoidosis-associated proteins.	(58)
**Ankylosing spondylitis**	Antigen array	ProtoArray	Serum	Reactivity against prefoldin subunit 5 (PFDN5) was identified in AS with uveitis, which was validated in mice model and apoptosis assay.	(9)
	Antigen array	NAPPA array	Serum	Multiple autoantibodies targeting toward connective, skeletal, and muscular tissue were strongly associated with AS patients.	(59)
**Primary Sjögren**’**s syndrome**	Antigen array	ProtoArray	Saliva	Array analysis elicited 24 potential autoantibody biomarkers that can discriminate patients with pSS from both patients with SLE and healthy individuals, 4 of which were confirmed with ELISA.	(60)
	Antibody array	Bead-based cytokine microarray	Tear fluid	Statistically significant upregulation of 8 and downregulation of 4 cytokines was observed in SS patients compared to controls. a significant inverse correlation (r<−0.7) with Schirmer strip readings.	(61)
**Primary biliary cholangitis**	Antigen array	HuProt Array	Serum	Six proteins were confirmed as novel PBC autoantigens with high sensitivities and specificities.	(62)
	Antigen array	Recombinant human protein microarray	Serum	Two autoantigens, SPATA31A3 and GARP, showed high reactivity with primary biliary cholangitis sera, containing or not anti-mitochondrial antibodies.	(18)
**Meniere**’**s disease**	Antigen array	ProtoArray	Serum	Eighteen candidate antigens were detected in patients with Meniere’s disease using array analysis, eight of which were exclusively found in the inner ear fluid of patients.	(63)
**Acute disseminated encephalomyelitis**	Antigen array	ProtoArray	Serum	Nine candidate antigens were identified among 16 ADEM patients, though no significant difference was found between anti-myelin oligodendrocyte glycoprotein (MOG) positive or negative groups.	(64)
**Autoimmune polyendocrine syndrome 1**	Antigen array	ProtoArray	Serum	Identification of transglutaminase 4 (TGM4) antibodies as a male-specific in APS1 patients which could lead to male subfertility.	(65)
**Neuromyelitis optica**	Antigen array	ProtoArray	Serum	Three novel autoantibodies other than AQP4 were detected in the serum of one patient with Devic’s neuromyelitis optica.	(8)
**Behcet disease**	Antigen array	HuProt Array	Serum	Two-phase based strategy was employed to find anti-CTDP1 antibodies as distinctive biomarker for BD patients.	(11)
**Autoimmune hepatitis**	Antigen array	HuProt Array	Serum	Two-phase based strategy was employed to identify autoantigen RPS20, Alba-like, and dUTPase as highly AIH-specific biomarkers.	(62)
	Antigen array	Recombinant human protein microarray	Serum	Microarray comprising 1626 human recombinant proteins selected in silico for being secreted or membrane associated protein identified totally 8 novel autoantigens associated with AIH in two studies.	(17, 66)
**Juvenile Dermatomyositis**	Antigen array	Small lab-made 80- antigens array	Serum	Significant association of reactivity against Ro, La, Sm, and proliferating cell nuclear antigen with serum IFNα activity were observed in JDM patients	(67)
**Idiopathic inflammatory myopathy**	Antibody array	Multiplex bead-based cytokine assay	Serum	A complex set of immune and inflammatory modulating cytokines are significantly up-regulated in patients with IIMs.	(29)
**Dilated cardiomyopathy**	Antigen array	Recombinant human protein microarray	Serum	Two subsets of proteins identified by high-content microarray were constructed into a small protein microarray, of which 26 IgG related and 6 IgG3 related autoantigens were predominantly found for DCM.	(15)
**Alopecia areata**	Antigen array	Recombinant human protein microarray	Serum	Eight autoantigens identified by protein microarray and confirmed by western blot were fabricated into a small disease-associated protein chip suitable for fast diagnosis.	(14)
**Celiac disease**	Antigen array	Phage display array	Serum	Thirteen CD-specific antigens were identified and further validated by protein microarray containing immunoreactive antigens from a cDNA phage-display library biopanned by CD patients’ sera.	(22)
**Kawasaki disease**	Antigen array	E. coli proteome array	Plasma	Of ~4200 *E. coli* protein, ~70 proteins shown to have high accuracy were purified to fabricate KD focus arrays for training and blind-testing.	(68)

## Development of Antigen Arrays

Antigen arrays are very popular and widely used in autoantibody screening, especially for AIDs. Various types of antigens, from a focused selection of disease associated candidate proteins/peptides to the proteome of some organelle or even species, could be immobilized on slides to prepare the antigen array and screen the autoantibodies in diverse patients. Joos et al. first introduced the idea of antigen microarray in research of AIDs, where eighteen well-known autoantigens related to SLE and other AIDs were deposited onto nitrocellulose membranes and examined in reaction with patient sera (69). Later, Robinson et al. built a 1152-feature Connective Tissue Disease (CTD) array including 196 putative autoantigens spotted onto glass slides (70). Both studies confirmed that protein microarrays are capable of higher sensitivity than ELISA. In recent years, similar multiplex approaches were developed to screen specific autoantibody biomarkers for various AIDs, such as rheumatoid diseases (71, 72), type 1 diabetes (73), lupus nephritis (74), multiple sclerosis (75, 76), systemic sclerosis (46), SLE (37, 77, 78), juvenile dermatomyositis (67), and Sjogren's syndrome (79). Importantly, most of these studies included dozens to several hundreds of autoantigens. In addition, a multiplexed, bead-based system was developed that is also suitable for array construction and detection for this application (79). However, only a few of the most well-studied autoantigens were used to construct this detection system for screening autoantibodies in patients' sera.

## Proteome Microarrays

In order to comprehensively analyze the interactions of biomolecules, such as protein-protein interactions, protein-small molecules interactions, and kinase-substrate interactions, MacBeath et al. developed the functional proteome array (80). Following the advent of this technology, Zhu et al. subsequently fabricated a yeast proteome chip containing 5,800 GST-tagged recombinant yeast proteins, and subjected it to screening of protein-drug interactions and detection of posttranslational modifications (81). The idea was later commercialized as ProtoArray® Human Protein Microarrays by Invitrogen™ (now under Thermo Fisher Scientific) and contained over 9,000 full-length human proteins. To date, the ProtoArray® Human Protein Microarrays have been widely used in identification of new biomarkers for many AIDs, including neuromyelitis optica (8), RA (42), primary Sjögren's syndrome (60), chronic renal disease (82), inflammatory bowel disease (52), type 1 diabetes (55), Meniere's Disease (63), autoimmune polyendocrine syndrome type 1 (65), acute disseminated encephalomyelitis (64), ulcerative colitis (83), SLE (33), and ankylosing spondylitis (9). In addition to the ProtoArray® Human Protein Microarrays, several new commercial proteome arrays have become available with additional protein probes using different expression systems. The HuProt™ Human Proteome Microarray V1.0 was designed by Zhu lab at Johns Hopkins School of Medicine and produced by CDI Laboratories around 2012, containing 16,368 recombinant GST-His-tagged human proteins expressed in *S. cerevisiae* (10), and the current v4.0 contains more than 21,000 unique proteins. To date, the HuProt array has been widely used to screen novel autoantigen biomarkers of AIDs, such as autoimmune hepatitis (84), SLE (34), primary biliary cirrhosis (62), neuropsychiatric lupus (35), multiple sclerosis (23), and Behcet disease (11). Another high-density commercial array is the IMMUNOME™ v4 Discovery Array, which was designed by Sengenics using more than 1,600 full-length human proteins, including kinases, signaling molecules, cytokines, interleukins, and chemokines. On the IMMUNOME™ v4 Discovery Array, all proteins are tagged with biotin carboxyl carrier protein (BCCP), which acts as a folding marker and solubility enhancer (12). McAndrew et al. applied the IMMUNOME™ array in autoantibody profiling with SLE serum samples (36, 85). Similarly, Poulsen et al. investigated the global reactivity of autoantibodies in RA patients (13). Additionally, Horn et al. constructed a special array constructed with large cohorts of disease-associated proteins, consisting of around 37,200 redundant, recombinant human proteins derived the *E. coli* expression system (14, 15). Then, serum profiling of alopecia areata and dilated cardiomyopathy patients were conducted by Beyer et al. to identify novel disease biomarkers. This approach was later commercialized into UNIarray® by Protagen Diagnostics and used to profile biomarkers in the cerebrospinal fluid of multiple sclerosis patients (16). Grifantini et al. selected 1,626 membrane-associated and secreted proteins from public resources to assemble a special microarray and screened biomarkers in autoimmune hepatitis and primary biliary cholangitis patients (17, 18, 66). The latest type of proteome array is SeroTag from Luminex Corporation. In this array, over 7,000 human proteins as antigens are immobilized onto bead-based suspension arrays, and it was used to screen novel biomarkers in SLE, SSc, and RA patients with promising results (45).

## Nucleic Acid Programmable Protein Arrays

An alternative way to produce protein probes on protein microarrays is *in situ* expression using cDNAs coding tagged recombinant proteins which are adjacently printed with the tag specific antibodies on a slide. Incubating with the *In Vitro* Transcription and Translation (IVTT)-coupled cell lysates allows cDNAs to synthesize the target proteins, followed by immobilization by the tag specific antibodies. For example, Sawasaki et al. developed a cell-free protein synthesis system using the eukaryotic translation apparatus of wheat seeds (86), and generated a protein microarray containing over 2,000 proteins. They used this array to screen autoantigens associated with rheumatoid synovitis and lupus nephritis (43, 87). To date, Nucleic Acid Programmable Protein Array (NAPPA) is perhaps the most famous protein microarray adopting this strategy, on which all captured proteins were C-terminal GST tagged (19). The popular commercial NAPPA array was designed and generated by the Protein Array Core of the BioDesign Institute, and it contains more than 12,000 human proteins (88). It can also be prepared according to a customer's requirements. NAPPA arrays have been employed to identify autoantibody biomarkers for several AIDs, including ankylosing spondylitis (59), juvenile arthritis (48), osteoarthritis (49), and type 1 diabetes (20, 57). In addition, because all the protein probes on the NAPPA array were translated by the IVTT-coupled cell lysates system instead of being expressed and subjected to high-throughput purification prior to immobilization on the array, this allows the NAPPA array to be quickly and easily produced, especially when using protein probes that are more difficult to express and purify with traditional methods. Also, NAPPA arrays provide a rapid method to prepare a pathogenic proteome array for analyzing the outbreak of infectious diseases or some AIDs where infection may contribute to their pathogenesis. Bian et al. developed a viral microarray containing 761 antigens from 25 viruses based on NAPPA and used it to study anti-virus antibodies in juvenile idiopathic arthritis and type 1 diabetes patients (56, 89).

## Pathogenic Proteome Arrays

Aside from NAPPA, pathogenic proteome arrays can be constructed using traditional expression systems and applied in the screening of the reactive antibodies in pathogen induced diseases. For instance, Chen et al. constructed a bacterial proteome microarray composed of 4,256 proteins encoded by the *E. coli* K12 strain and subjected it to the investigation of biomarkers for inflammatory bowel disease (53). Meanwhile, Kuo et al. created an *E. coli* proteome microarray with around 4,200 proteins and analyzed the antibody spectrum in pre-eclamptic women (90) and patients with Kawasaki disease (68). Loebel et al. also employed an EBV-derived peptide microarray to profile the serological response in chronic fatigue syndrome (51). Furthermore, phage display libraries can provide an alternative method for protein microarray construction. Talwar et al. created an T7 phage display immune-cell cDNA array that was bio-panned to obtain 1152 potential sarcoidosis antigens, and a microarray was constructed to test the sera from sarcoidosis and tuberculosis patients (21). A similar method was adopted by D'Angelo et al. in profiling celiac disease antibodies (22).

## HPA Protein Fragment Arrays

The Human Protein Atlas (HPA) is an antibody-based proteomics database containing abundant of information about protein expression and localization in various cells and tissues, under both normal and diseased conditions (24). In order to select each protein's unique fragments, Protein Epitope Signature Tags (PrESTs) were developed by Berglund et al. (91). The HPA protein fragment arrays were constructed using these PrEST fragments, including over 42,000 unique purified fragments deriving from ~20,000 human proteins (23). Compared with the HuProt™ Human proteome microarray, the HPA protein fragment array has demonstrated better performance because they have much fewer off-target interactions. Therefore, the HPA protein fragment array is a valuable method to identify autoantibody targets with higher specificity in various autoimmune disease (23). Using similar strategy, Uhlen et al. produced an array containing 11,520 protein fragments with planar or bead-base methods and subjected them to antibody screening in multiple sclerosis (40, 92). Additionally, PrEST arrays of different sizes were also applied for osteoarthritis, sarcoidosis, and SLE patients (25, 49, 58).

## Peptide Microarrays

In addition to protein microarrays constructed using full-length or truncated proteins, peptide arrays have also been developed using hundreds to thousands of peptides that were pre-synthesized or in situ synthesized directly on a surface. To date, peptide arrays have been applied in screening autoantibodies in AIDs, various cancers, and other diseases. Compared with full-length proteins, peptides are much cheaper, more stable, and allow for a higher capability of modification, such as acetylation or methylation. Generally, the high density peptides on the array are *in situ* synthesized using a semiconducting photolithography approach (93). The RepliTope™ Peptide Microarray, containing large random-sequences, was reported to identify potential peptide biomarkers in patients with lupus and determine its central nervous system (CNS) manifestations (26). In addition, peptides derived from the specific epitopes of disease-related proteins could be used to fabricate a disease-specific microarray to investigate antibody response at up to single-amino acid resolution. For instance, Haddon et al. developed a silicon-based peptide microarray, composed of over 5,700 features corresponding to 843 unique peptides derived from the U1-70K protein, and applied it in screening specific autoantibodies in SLE (38). Hecker et al. employed 3,747 peptides deduced from 45 candidate proteins to construct a high-density peptide microarray, and performed the analysis of IgG autoantibody reactivity in serum and cerebrospinal fluid of multiple sclerosis (41). Recently, Li et al. constructed a peptide array, including 73 potential antigenic epitopes in 14 autoantigens and subjected it to evaluate potential diagnostic values for each epitope in SLE patients (39).

## Antibody Microarrays

In addition to autoantibodies, some other dysregulated biomolecules in disease progression, such as cytokines, chemokines, growth factors, and mRNAs, are considered reliable sensors of AIDs. Sandwich ELISA is a well-developed and popular tool in semi-quantitative or quantitative measurement of target molecules using high quality antibodies. Following need for more monoclonal antibodies with high-affinity and specificity, antibody arrays were constructed to test hundreds of targets simultaneously and greatly expanded the detection of target objects. In particular, the cytokine antibody arrays are extensively used in the field of AIDs, because the cytokines are not only indicators but also therapeutic targets of these diseases (94). One of these earliest arrays was designed by Kader et al., and it was used to investigate 78 different cytokines and growth factors in pediatric patients with IBD (95). A similar approach was also adopted for use in other diseases such as SLE, RA, and MS (96). In recent years, a series of commercial antibody arrays was developed by RayBiotech, including human biomarkers, cytokines, inflammatory factors, and growth factors. For AIDs, the RayBio arrays were successfully applied in various diseases, including Crohn's disease (27), preeclampsia (50), and SLE (28, 97, 98). More recently, incorporation of anti-cytokines antibodies into multiplexed bead-based assays is becoming increasing popular, and this type of analysis has been performed in several different AIDs such as idiopathic inflammatory myopathy, RA, and Sjogren's Syndrome (29, 44, 61, 99). Currently, most of antibodies on the array are well-characterized monoclonal antibodies. However, recombinant antibodies are also explored and applied to construction of antibody microarrays. Carlsson et al. fabricated scFv microarrays targeting immune regulatory proteins and applied them in serum profiling of SLE and SS patients (47). Apart from studying cytokines, antibody arrays could also be used to measure different antigens involved in immune dysregulation. For instance, Lin et al. utilized an antibody array that consists of 82 mouse monoclonal antibodies against human cluster of differentiation (CD) antigens to investigate leucocyte reaction for SLE diagnosis (30). A potential disadvantage of antibody microarrays is the specificity of the antibodies with more abundant off-target proteins (100). Thus, acquisition of high-quality antibodies with high affinity and specificity are essential for biomarker identification.

## Lectin Arrays

Considering over half of proteins are glycosylated *in vivo*, the study of glycoproteomic alteration under different physio-pathological states may contribute to the discovery of disease associated biomarkers (31). Lectins, a group of carbohydrate-binding proteins specific binding different glycans could be used to assemble the lectin microarrays to profile glycosylation in tissues. Regarding AIDs, lectin arrays have been applied to identify and monitor glyco-biomarkers in IBD and RA (32, 54). Hopefully in the future, the progress of lectin microarray technology may contribute to its broader applications in AIDs.

## Key Issues of Protein Microarrays in Aid Biomarker Discovery

Although a number of review papers have been published on protein microarrays, where general illustrations of their classifications, experimental procedures, and applications could be found (5, 7, 24, 101, 102), it still seems to be rare to find a comprehensive discussion on the key issues when using protein microarrays in AID biomarker discovery. Such issues can include biosample collection, optimization of experimental design with consideration for minimized cost, choice of different protein arrays, data analysis, novel biomarker validation, and even the technical transference to clinic lab tests. Addressing each of the challenges would be helpful in improving the application of protein microarrays in future studies.

## Bio-Sample Collection

Sample collection is the first and most critical step in the identification of AID biomarkers using protein microarrays. Ideally, patients with clinical manifestations specific to one AID would be recommended for protein microarray experiments. However, AIDs could be complicated in terms of pathogenesis and progression, and some patients may encounter more than one disease. Moreover, age, gender, population, and other demographic variables may further contribute to the heterogeneity of patient groups, which might elicit contradictory results across different studies.

For some well-characterized AIDs, a key consideration is sample stratification. Patients could be grouped by multiplexed AID manifestations, primary or secondary pathogenesis, distinct immunophenotypes, etc. based on previous literature, and the discovery of protein microarrays may in turn assist in disease subclassification (103, 104). Another consideration is the sampling time. Longitudinal prospective studies using certain biomarker panels could be applied in disease prevention, activity monitoring, as well as prognosis following treatments (105–107). Furthermore, additional large cohorts of samples should be collected to prove any novel discoveries. For some AIDs with low incidence rates, on the other hand, sample collection is often driven by availability. Thus, collaboration between several large medical institutes or hospitals is strongly recommended to obtain sufficient samples under consistent criteria. Usually, researchers are inclined to make use of all available patients, which means the samples used in the development stages were recruited in validation assays.

In addition to serum samples, which are the most popular sources used to screen auto-antibodies for AIDs, other samples such as urine, cerebral fluid, or other body fluids could be collected, as distinct sets of biomarkers might exist corresponding to differentially impaired organs (35, 43, 58, 61).

## Experimental Design for Cost-Effective Protein Microarray Assays

Considering the high cost of a commercial protein microarray, especially a proteome microarray containing tens of thousands of recombinant protein probes, experimental design with consideration for cost-effectiveness would be necessary for a screening assay with a large cohort of samples. A design with two-phases has more recently attracted researchers' attention (84). In phase I of this design, a small collection of samples was randomly selected for a screening assay with a high-density proteome array to identify candidate biomarkers with relative loose criteria. While in phase II, these candidate probes were used to construct a disease focused mini array, which contains more than ten blocks on one slide and can be a cost-effective way to evaluate samples in large cohorts. To date, this strategy has been adapted to a variety of different studies (11, 35, 62, 108). In addition, some scientists have performed this screening assay using mixtures of several samples with similar conditions on limited proteome arrays (70). However, the results from mixture samples might produce a higher possibility of losing information on targets with lower affinity or titers.

## Considerations in Design of Protein Microarray Experiments

Various aspects of protein microarrays, such as the protein probes utilized, the chemical modifications on the microarray surface, and the fabrication and detection methods, could impact the result and even the transition to clinical experiments regardless of whether the array is lab-made or commercial (109–112). As mentioned above, both full-length proteins and peptides can be supplied as the primary materials for protein microarrays. Generally, full-length proteins are expected to remain in their original *in vivo* structure in order to mimic the authentic interaction with autoantibodies or other molecules (113–115), while peptides could provide much more information to elucidate specific binding epitopes (116–120). Thus, it might be reasonable to use full-length protein arrays for initial narrowing (phase I) of the target antigens, and then adopt a more cost-effective synthetic peptide arras to further evaluate the binding sites (phase II), though this strategy might require collaboration between several institutes or companies. On the other hand, a self-developed array could focus the antigens associated with the specific disease [e.g. and choose some pathogen antigens, especially in the assay with microbiome-related AIDs (89)]. In addition to antigen arrays, antibody arrays are more attractive in profiling dysregulated cytokines or other known disease-related molecules in serum or other samples in a quantitative fashion (121–124). Meanwhile, when considering glycosylated target molecules, lectin arrays could bring the knowledge of autoantibody-autoantigen interactions to a new level (125, 126). However, extending the scope of the probes, including proteins or peptides with various post-translational modifications or aptamers, could refine and increase the range of identified biomarkers (24). For instance, the affinity-based slow off-rate modified aptamer (SOMAmer) technology, which has been applied in proteomic profiling across various diseases and health states, could also be viable for AIDs (127).

In addition, the properties of slide surface are critical to the performance of protein probes. Achieving a high-density, robust immobilization method while retaining proper folding and orientation of the protein are the common requirements for various slides. Covalent (i.e., SuperEpoxy Slide) or noncovalent (i.e., FAST^®^ and PATH^®^ Slide) chemistries are both available for commercial protein microarray slides. Besides the planar or bead-based surface, a 3-dimensional matrix on a glass surface would help to maintain the proteins structure and also support additional space for immobilizing more proteins (102).

Fluorescent dyes and radioisotopes are two common regents in detection of binding activities on a protein microarray. Fluorescent dye labeled reagents enable reliable detection with high signals and low background. However, fluorescent labeling may interrupt protein structure and even its activities, which could impact the results of the array. Though radioisotope labeling has overcome this fault, this kind of labeling results in much higher background signals. Novel label-free detection approaches such as surface plasmon resonance, carbon nanotubes, reflective phantom interface, etc. are expected to address the problem with greater sensitivity (101).

## Data Analysis and Novel Biomarker Validation

A general outline of data analysis for identifying biomarkers using protein microarray involves data acquisition, pre-processing, bioinformatic analysis, and differential target selection (128). To narrow down the scope of targets for further validation, data processing and mining are crucial in identifying biomarker candidates, especially when dealing with large amounts of raw data from high-density proteome microarrays. Commercial human proteome microarrays usually entail a respective processing method, such as ProtoArray Prospector Software for ProtoArray®. Most methods have been adopted from a similar one that is used in DNA microarray technology. A consensus bioinformatics procedure has still not been achieved among researchers working on protein microarrays. With regard to biomarker discovery, retrieving a suitable amount of biologically meaningful biomarkers demands special feature selection methods (129). Computational algorithm analysis could facilitate selection of appropriate biomarker profiles with better predictive accuracy (30). For data sharing, online databases such as Protein Microarray Database (130), AAgMarker 1.0 (131), and AAgAtlas 1.0 (132) offer a comprehensive platform to overview data from protein microarrays as well as various candidate autoantibody biomarkers in different diseases. To validate the identified novel biomarkers, traditional methods such as ELISA and immunoblotting would be preferred because of their higher compatibility with the current popular methods in lab tests in hospitals. Success with validation means there would be a much higher probability of technology conversion and the following industrial manufacture in the downstream.

## Conclusion

Autoimmune diseases are characterized by elevated local or circulatory autoantibodies, cytokines, chemokines, etc., which are usually present before the onset of disease and might be associated with various clinical manifestations. Thus, screening and identifying disease indicators is critical for diagnosis, evaluation, as well as prediction of the disease risks. Generally, the identification of more biomarkers would contribute to increased accuracy in determination of patient diagnosis. The application of proteome microarrays has drawn growing interest in identification of new biomarkers for AIDs because of its outstanding performance in numerous studies. Protein microarrays are quick, cost-effective, high-throughput, and high-sensitivity, proving them to be a powerful technology in basic and translation research. With the rapid technical developments and accumulation of various biomaterials, increasing types of protein arrays, including lab-made disease focused antigen microarray, versatile cytokine microarray, and high-density proteome microarrays, were applied to screen and identify biomarkers for various AIDs. In the foreseeable future, more less-studied AIDs are likely to be examined for novel biomarkers using protein microarray approaches.

Despite numerous successful application of protein microarrays in AID biomarker discovery, there are still challenges that need to be overcome in order to improve the efficiency of these assays, including, but not limited to, the establishment of standard data analysis pipelines and the transition of novel discoveries to clinical lab tests. Hopefully, integration of new computer methods, new biomaterials and artificial intelligence with the protein microarray technology will shed light on biomarker discovery for AIDs in the future.

## Author Contributions

JZ and CH designed the research. SL, YB, and NS collected and analyzed the data. SL and GS wrote the paper. JL edited the manuscript. All authors contributed to the article and approved the submitted version.

## Funding

This study was supported by the National Natural Science Foundation of China (81771780), the National Key Research and Development Program of China (2019YFC0840603, 2017YFC0907601, and 2017YFC0907602), and the CAMS Initiative for Innovative Medicine (2017-I2M-3-001 and 2019-I2M-2-008).

## Conflict of Interest

The authors declare that the research was conducted in the absence of any commercial or financial relationships that could be construed as a potential conflict of interest.
